# Analysis of Saliva Composition: Parathyroid Hormone-Related Protein, Total Protein, and Secretory Immunoglobulin A (sIgA) in *Rattus norvegicus* with Stunted Growth

**DOI:** 10.1055/s-0042-1755558

**Published:** 2022-10-11

**Authors:** Udijanto Tedjosasongko, Sindy Cornelia Nelwan, Soegeng Wahluyo, Mega Moeharyono Puteri, Ardianti Maartrina Dewi, Retno Pudji Rahayu, Ilvana Ardiwirastuti, Puspita Ayuningtyas, Regina Ayu Pramudita, Aisyah Marwah

**Affiliations:** 1 Department of Pediatric Dentistry, Faculty of Dental Medicine, Universitas Airlangga, Surabaya, Indonesia; 2 Department of Oral Pathology, Faculty of Dental Medicine, Universitas Airlangga, Surabaya, Indonesia

**Keywords:** stunting, PTHrP, sIgA, total protein

## Abstract

**Objective** This study aimed to determine total protein, secretory immunoglobulin A (sIgA) and parathyroid hormone-related protein (PTHrP) levels in the saliva of rats with stunted growth.

**Materials and Methods** Experimental laboratory research with a pre-and posttest control group design was conducted. Seventeen albino rats ( *Rattus norvegicus* ) were divided into the control group (eight rats) and the treatment group (nine rats). Rats in the treatment group were exposed to aflatoxin B1 5µg/kg orally for 5 weeks. Anthropometry data (body length, body weight) and saliva of *R. norvegicus* were collected. The levels of PTHrP and sIgA in the saliva were measured using an enzyme-linked immunosorbent assay kit for rats and the Bradford test for total protein and analyzed using SPSS 25.0.

**Results** Aflatoxin caused stunted growth in rats in the treatment group. There was a significant difference in body length, salivary flow, PTHrP, sIgA, and total protein in the treatment group compared with the control group. The average rat's body length change in the control group was 6.4 ± 1.1mm/5 weeks, while in the treatment group, the change was 3.7 ± 0.9 mm /5 weeks. There was no significant weight gain in the treatment group compared with the normal group. The average values of PTHrP, sIgA, and total protein in the control group were x̄0.9, x̄18, and x̄0.7 m./L, respectively, while in the treatment group, they measured x̄0.4, x̄10.7, and x̄0.5 mg/L, respectively.

**Conclusion** This study showed that salivary flow, PTHrP, sIgA, and total protein levels in the saliva were significantly lower in stunted rats compared with normal rats.

## Introduction

Caries and stunting are major health problems in Indonesia. According to the 2018 National Health Research (RISKESDAS), the prevalence of caries in Indonesia is high, affecting 45.3% of the population. 1 Stunting impacts 22% of children under the age of five globally. Southeast Asia has the highest rate of stunting worldwide (34.4%), where one in every three children under the age of five has stunted growth. 2 Indonesia is one of four Southeast Asian countries with the highest rates of stunting, 3 with a prevalence of 31.8%. Dental caries are caused by multiple factors, including an increase in pro-inflammatory cytokines, a decrease in the amount of saliva and changes in the saliva composition. 4 Such changes can be triggered by conditions associated with malnutrition, such as stunting. 5

Stunting has multiple causes, including heredity, inadequate breastfeeding, inadequate nutritional intake (macro nutrition and micronutrients), hormonal disorders, infectious diseases, poor water sanitation hygiene, poverty, and an unhealthy lifestyle. This condition causes environmental enteric dysfunction (EED) and protein energy malnutrition (PEM). 6 7 If stunting conditions are not prevented in the first 1,000 days of life, there is a risk of linear bone growth disorders and height attention deficit (HAD) in adults and adolescents. 8

PEM is caused by an imbalance between dietary energy intake and the physiological energy expenditure required by every cell in the body. In PEM, disruptions in the maintenance of the availability of energy for basal cell metabolism and nutrients can impair tissue synthesis and tissue repair, and physiological dysregulation of the endocrine system. 9 10 PEM and EED can disrupt the process of endochondral ossification, fibroblast proliferation and growth hormones, causing growth hormone (insulin-like growth factor-1), thyroid hormone and parathyroid hormone (PTH) to be suppressed and resisted. 11

The condition of the oral cavity is also affected by stunting. 5 Salivary gland hypofunction and reduced salivary flow are caused by EED and PEM in stunted children. 12 Compared with normal children, children with stunted growth have a higher incidence of dental caries, reduced salivary flow, and delayed tooth eruption. Dysfunction of the salivary glands can cause decreased saliva flow in stunted children. 13 14 Dysfunction of an organ is caused by a decrease in the organ's basal metabolism. Deficiencies in energy and protein play a role in this dysfunction. 9 The total serum protein in children with stunted growth can be calculated to assess protein levels. 15 The whole composition of saliva, including levels of sIgA, total protein, and PTHrP, may alter as a result of these situations. Dental caries occurs due to a reduction in antibody function. The antibody immunoglobulin A, which is released in saliva as secretory immunoglobulin A (sIgA), helps prevent caries. Reduced sIgA levels in stunted children can lead to caries because the reduced amount affects the function of blocking bacterial binding, colonization, and bacterial metabolism disturbance. 16 Tooth eruption is influenced by various factors, including parathyroid hormone-related protein (PTHrP), which act as a signal between hormones in the dental follicle. 17 Serum and saliva analysis can identify hormone levels in children with stunted growth. A decrease in PTHrP indicates the rate of oral cavity growth and development and the rate of eruption. 18

Measuring total protein, sIgA, and PTHrP in the blood as a means of determining stunting is an invasive method. Using saliva is easier and less invasive compared with taking blood samples. In addition, saliva has long been recognized that its ultrafiltration from serum. 19 Salivary composition analysis will be performed using an animal model as a form of initial research. The *Rattus norvegicus* was chosen as the study's experimental animal since it is one of the larger rodents that can be used in stunting experiments. It has also been used in previous studies. The biggest rats were selected to take into consideration the maximum amount of saliva stimulation and saliva production that can be taken to analyze. Aflatoxin substances will be exposed to animal models ( *R. norvegicus* ). Previous research has proven that exposure to aflatoxin substances induces stunting in both humans and rats. 20 This study aimed to determine total protein, sIgA, and PTHrP levels in the saliva of rats with stunted growth. For further study, salivary composition could be used to predict stunting in children. The caries rate in stunted children can be assessed by using sIgA analysis. Also, growth and development of the child's oral cavity can be assessed using total protein and PTHrp.

## Materials and Methods

Experimental laboratory research with a pre- and posttest control group design was conducted. The research was conducted at the Universitas Airlangga Research Center Laboratory, Faculty of Dental Medicine with the ethical research clearance assessment (No 536/HRECC.FODM/IX/2021). Seventeen albino rats ( *R. norvegicus* ) aged between 3 and 4 weeks were divided into treatment group and a control group.

### Stunted Rat Modeling

Nine *Rattus norvegicus* were used in each group (treatment and control group). One rat died during the research process, leaving eight in the control group and ten in the treatment group. The anthropometry of both groups (body length and body weight) was measured under isoflurane vaporized inhalation sedation. Body length was measured from farthest the point of the tip of the nose to the tip of the anus with a digital caliper, and body weight was measured using a digital scale. The treatment group was exposed to aflatoxin B1 (AFB1) (FERMENTEK Ltd 4 Yatziv street, P.O.B. 47120, Jerusalem 9147002, Israel) 5µg/kg orally for 5 weeks to induce stunted growth. The dose for each rat is calculated using the rat's weight. Oral intubation was used to give powder AFB1 (1.25mg/mL) dissolved in dimethyl sulfoxide solution. The suspension was administered orally, around 5 mL with a 20-gauge intubation needle and monitored for 5 weeks. Anthropometric measurements were taken following an experiment in the rats' fifth week of life. 18

### Saliva Collection

Stimulated saliva was collected by injecting 0.5 mg/kg intramuscular pilocarpine (Cendo Carpine from PT. Cendo Pharmaceutical Industries, Cisirung No. Km 67, Bandung, 40256, West Java, Indonesia). The stimulated saliva collection was conducted in the sublingual cavity of the oral cavity for 5 to 15 minutes with a micropipette. Saliva was stored in sterile tubes in a styrofoam box with an ice bath at −4°C for transfer, then centrifuged at for 10 minutes, and the saliva was stored in the freezer at −80°C.The collected saliva was then centrifuged and analyzed. 21

### PTHrP, sIgA, and Total Protein Measurement

PTHrP levels were measured using the rat PTHrP ELISA Kit (PT Biozatix Indonesa, Griya Agung Street No. 59, North Jakarta, Indonesia, with catalogue number DZ08184330-EB). The Rat sIgA enzyme-linked immunosorbent assay (ELISA) kit (PT Biozatix Indonesa, Griya Agung Street No. 59, North Jakarta, Indonesia, catalogue number DZ08185320-EB) was used to measure the level of sIgA. The ELISA procedures were following ELISA kits manufacturers' protocols. While the Bradford test was used to calculate the total protein, the test was conducted using Merck KGaA, Darmstadt, Germany with catalogue number P0834–10 × 1 mL.

Measurement of PTHrP and sIgA levels was done using ELISA. Briefly, 100 µL of saliva was put into each well plate and then incubated for 90 minutes at 37°C. Samples were added to 100 µL of biotinylated detection rat's antibody and incubated for 60 minutes at 37°C and aspirated. The next step streptavidin-HRP was added to each well plate and incubated for 30 minutes at 37°C. Ninety µL of 3,3',5,5'-Tetramethylbenzidine (TMB) substrate solution was added and incubated at 37°C for 15 minutes before adding 50 µL of stop solution. The sample solution was then read out on an ELISA reader with a wavelength of 450 nm. The result is read as an absorbance value (optica density), as a form of absorbance unit used as a standard for PTHrP and sIgA levels. 22

Measurement of total protein content began with centrifugation of the sample at 3000 rpm for 5 minutes. Saliva was taken with a micropipette as much as 10µ and placed on a block of fluid blocks, and one block was added as a control. The 10 µL saliva samples were then mixed with 200 µL of Bradford's reagent standard solution and the sample was placed on a microplate using a multichannel pipette. After 30 seconds of mixing with a plate shaker, the solution was incubated at room temperature for 10 minutes. The total protein concentration was determined by observing the return of brown to blue. The absorbance of the color change was then examined on a microplate reader with a wavelength of 595 nm. The total protein concentration was determined using the Bradford assay. 23

### Statistical Analysis

The obtained results were statistically analyzed using an independent *t* -test to assess differences in body length, volume saliva, PTHrP levels, sIgA levels, and total protein levels between treatment and control groups. The Wilcoxon sign rank test was used to analyze differences in body length change, because the distribution was not normal in the control group. The level of significance was set at *p* *<* 0.05. The Shapiro–Wilk test was used to assess normality, and Levene's test was used to assess the homogeneity of variance. The statistical analysis was performed using IBM SPSS Statistics version 25.0 (IBM Corp., Armonk, NY, United States) for Windows, released 2018 in New York.

## Results

**Fig. 1:**
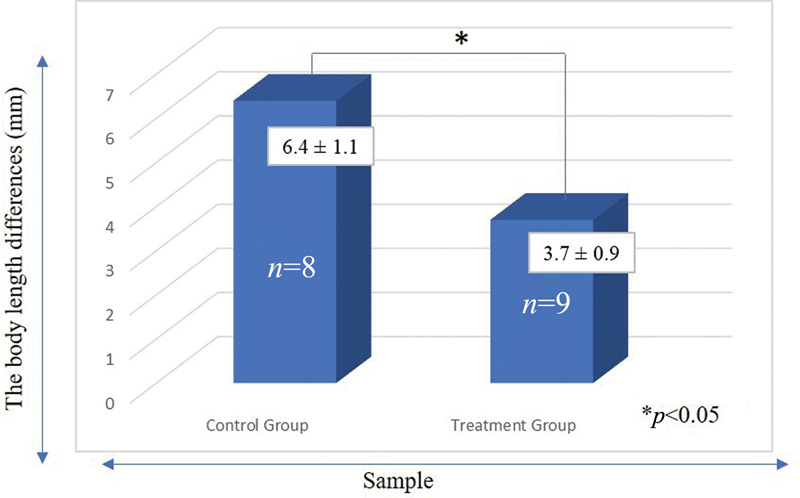
The differences in length of rats in the control groups and treatment groups. *Indicated significantly difference between groups.

**Fig. 2:**
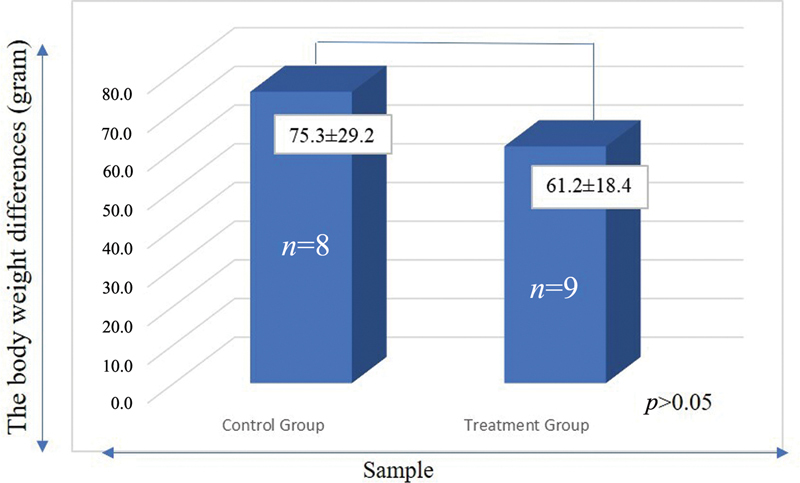
The differences in weight of rats in the control groups and treatment groups.

**Fig. 3:**
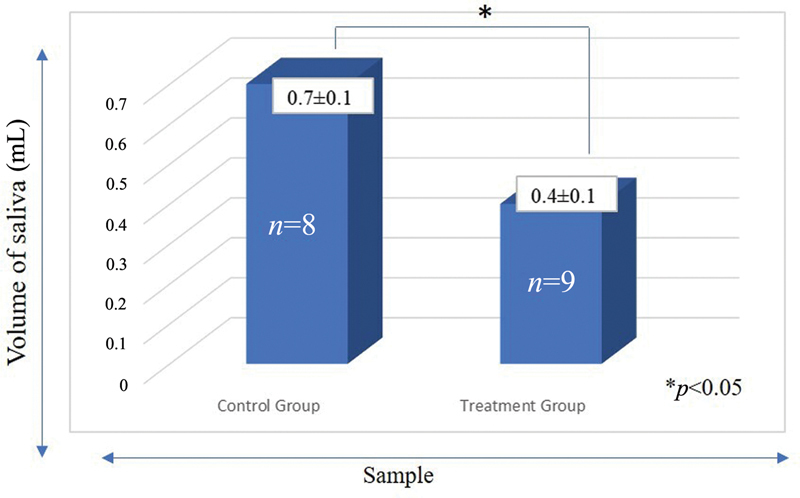
The salivary flow differences of rats in the control groups and treatment groups. *Indicated significantly difference between groups.

Based on statistical analysis between control and treatment group of 5 weeks rats showed that the body length gain in the treatment group (3.7 ± 0.9 mm) was significantly ( *p* *<* *0.* 05) lower than the control group (6.4 ± 1.1 mm) ( Fig. 1 ). On the other hands, the results of body weight gain showed there was no significantly difference. The average body weight gain in the treatment rats was 61.2 ± 18.4 g, while in control rats, the average body weight gain was 75.3 ± 29.2 g ( Fig. 2 ). In salivary volume analysis, the results showed on decreasing in salivary volume significantly ( *p* *<* 0.05 *)* between treatment group (0.4 ± 0.1 mL) and control group (0.7 ± 0.1 mL). The stunted rats (treatment group) had a lower volume saliva compared with the normal rats (control group) ( Fig. 3 ). These results indicated that under stunting condition decreased body weight, body length, and salivary volume compared with healthy condition.

**Fig. 4:**
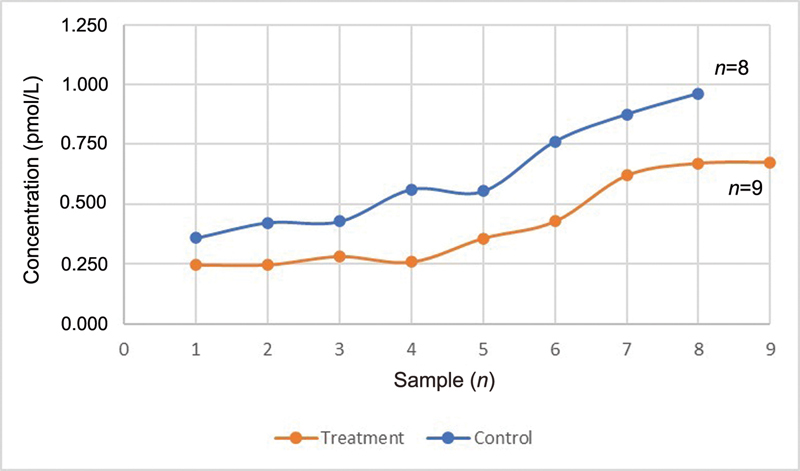
The difference in average parathyroid hormone-related protein between the control and treatment groups.

**Fig. 5:**
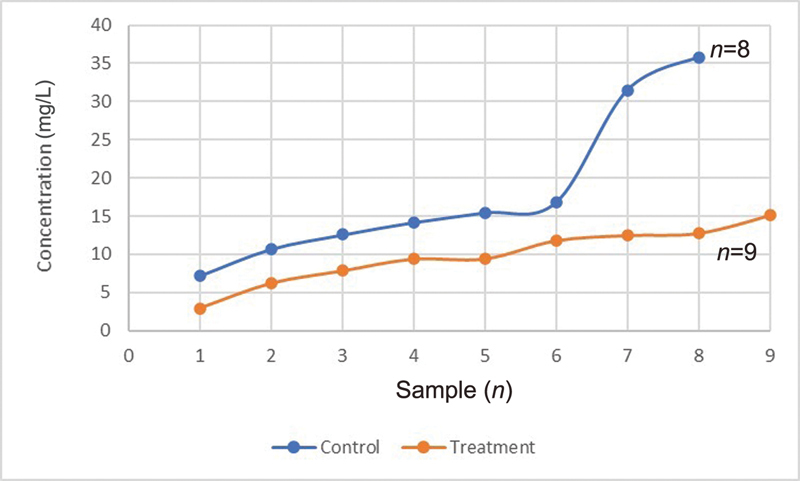
The difference in average secretory immunoglobulin A between the control and treatment groups.

**Fig. 6:**
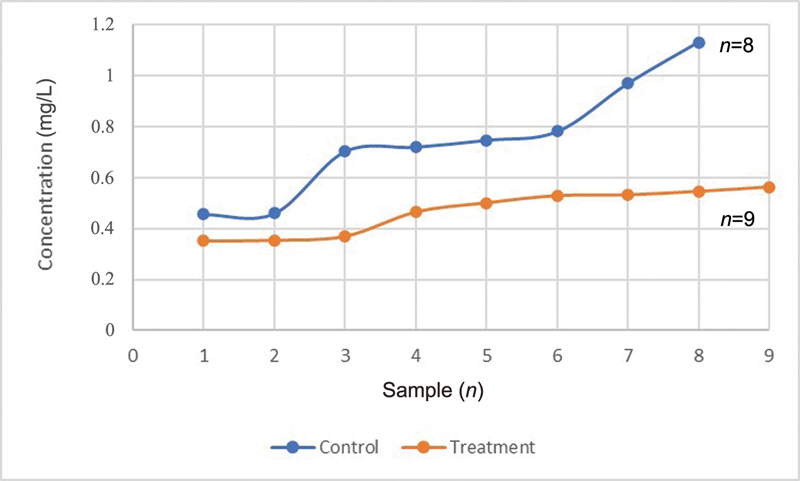
The difference in average total protein concentrations between the control and treatment groups.

Based on statistical analysis, PTHrP levels, sIgA levels, and total protein levels in saliva treatment group were significantly lower ( *p* *<* 0.05) than control group. In the control group, the average values of PTHrP, sIgA, and total protein were 0.9 pmol/L, 18 g/mL, and 0.7 mg/L, respectively. In the treatment group, PTHrP, sIgA, and total protein levels were 0.4 pmol/L, 10.7 g/mL, and 0.5 mg/L, respectively ( Figs. 4 , 5 , 6 ).

## Discussion

Aflatoxin is a toxic chemical produced by metabolism of the fungi *Aspergillus flavus* and *A. parasiticus* , which are commonly found in tropical and subtropical climates and occur in corn, spices, nuts and in the milk of animals that feed on these foods. AFB1, aflatoxin B2 (AFB2), aflatoxin G1 (AFG1), and aflatoxin G2 (AFG2) are the four principal kinds of aflatoxin compounds, with AFB1 being the most toxic. These substances are processed in the liver and are carcinogenic, with the ability to induce hepatocellular carcinoma. 24 Aflatoxin can also cause gastrointestinal disease, diarrhea, immune system problems, and problems with growth and development. This is because aflatoxin disrupts the microflora balance in the intestine, promoting intestinal inflammation, disrupting the villi in the intestine, and interfering with carbohydrate, fatty acid, amino acid and vitamin absorption and metabolism. 23 This is supported by Zhou et al, who showed that rats exposed to AFB1 at various doses exhibited gastrointestinal problems and stunting. 18 Children's growth is also stunted by the effect of AFB1. This was confirmed by Alamu et al, who demonstrated elevated serum levels of AFB1 in children with stunted growth. 25 As the mechanism and effects of aflatoxin are similar to the etiological incidence of stunting in children in underdeveloped countries with tropical climates, we chose to use aflatoxin to elicit stunted growth in the rats in our study. There was a 0.7 ± 0.9 mm 5 weeks difference in body length between the treatment and control rats, as well as a significant body length difference between the two groups, with the treatment rats experiencing growth problems and subsequent stunting.

PEM can be caused by disturbances in the absorption and metabolism of macronutrients and micronutrients in the intestines, which result in an imbalance in carbohydrate and protein intake. Clinical disorders, such as kwashiorkor and marasmus, are caused by acute or severe PEM, whereas long-term PEM causes stunting. 9 Immune system disorders, gastrointestinal disorders, cardiovascular disorders, respiration disorders, neurology disorders, hematology disorders, and PEM accompanied by micronutrient deficiencies (calcium, phosphate and vitamins A, C, and D) can interfere with the odontogenesis process, increasing the risk of caries, delayed eruption, and impaired salivary glands. 11 26 The results showed that rats exposed chronically to AFB1 cause shorter body length compared with control rats. PEM conditions that occurred in the intervention rats were accompanied by decreased levels of total protein in the saliva, hormonal abnormalities, including decreased levels of PTHrP and decreased antibodies in salivary secretions (sIgA).

The difference in total protein levels between the treatment and control groups was significant in this study ( *p* *<* 0.05), indicating protein deficiency and the presence of PEM in the rats with stunted growth. The total protein was measured to determine the total quantity of protein in the serum and plasma cells, with a 60 albumin and 40% globulin composition noted. Globulin and albumin are made in the liver and are commonly used to evaluate malnutrition. Serum albumin decreases in the event of an increase in systemic inflammation, followed by an increase in interleukin-6 and tumor necrosis factor-cytokines, reduced liver function, nephrotic syndrome, and protein-losing enteropathy through the gastrointestinal system. 2 According to Abdullahi et al 26 and Raval et al 13 , there is a decrease in serum total protein and albumin in undernourished children, which is consistent with our findings of a decrease in total protein in the saliva. 15 27 This suggests a reduction in total protein, which is also reflected in the composition of the saliva.

Changes in saliva composition in rats with stunted growth were also observed in relation to sIgA levels. The salivary glands release sIgA, which is an immunoglobulin. The stunted rat group experienced a significant decrease ( *p* < 0.05) in sIgA due to thymus gland atrophy and increased systemic inflammation. PEM conditions and deficiencies of micronutrients, such as zinc, magnesium, and phosphate, which are required for thymus gland growth, cause thymus gland atrophy. Micronutrient deficiency has a negative impact on the size of the thymus. 28 Meanwhile, PEM causes salivary gland hypofunction and results in decreased adrenoceptor density and salivary flow. PEM further decreases the production of immunoglobulins in the saliva of stunted rats, particularly sIgA, as was evident in our study.

We noted a significant decrease in salivary PTHrP ( *p* < 0.05) in the treatment mice. This is consistent with Gentile and Chiarelli, 16 who showed that a decrease in serum PTHrP correlated with stunted growth. 18 Decreases not only occur in the serum but are also found in salivary PTHrP as an illustration of serum. PTHrP causes stunting because PTHrP's function is to maintain chondrocyte hyperplasia in endochondral ossification so that bone width can be maintained. 29 PEM in stunting causes gland hypofunction and suppresses growth hormone release through chronic systemic inflammation. 29 Some rats in the stunting group in this study gained weight. Differences in body weight before and after treatment between the control and treatment groups were identified, but the difference was not significant ( *p* > 0.05). Stunted children are more likely to gain weight and become overweight. Hajri et al and Al-Taiar et al showed that stunted children are at risk of overweight condition. 30 31 EED, liver damage, and vitamin D deficiency in stunted conditions are all contributing factors. 8 A decrease in serum 25 hydroxyvitamin D(25(OH)D) is caused by vitamin D deficiency. Vitamin D controls adipogenesis in adipose tissue and plays a role in glucose and lipid metabolism. As vitamin D is required for bone growth homeostasis, a deficiency of the vitamin causes stunted growth. Obesity results from inadequate adipogenesis, which causes fat deposits in cells, tissues, and the subcutaneous tissue. 32

## Conclusion

This study showed that salivary flow, PTHrP, sIgA, and total protein levels of saliva were significantly lower in rats with stunted growth compared with normal rats.

## References

[1] Riset Kesehatan Dasar (RISKESDAS) No Title. Has Utama Ris Kesehat Dasar 2018. Published online 2018. https://kesmas.kemkes.go.id/assets/upload/dir_519d41d8cd98f00/files/Hasil-riskesdas2018_1274.pdf. Accessed July 3, 2022

[2] Keller U Nutritional laboratory markers in malnutrition J Clin Med 2019 8 06 E775 10.3390/jcm8060775 PMC6616535 31159248

[3] WHO Title levels and trends in child malnutritionPublished online 2020. https://www.who.int/publications-detail-redirect/9789240025257. Accessed July 3, 2022

[4] Folayan M O Arije O El Tantawi M Association between early childhood caries and malnutrition in a sub-urban population in Nigeria BMC Pediatr 2019 19 01 19 21 31722683 10.1186/s12887-019-1810-2 PMC6852898

[5] Achmad H Ramadany S A review of stunting growth in children: relationship to the incidence of dental caries and its handling in children Syst Rev Pharm. 2020 11 06 230 235

[6] Danaei G Andrews K G Sudfeld C R Risk factors for childhood stunting in 137 developing countries: a comparative risk assessment analysis at global, regional, and country levels PLoS Med 2016 13 11 e1002164 10.1371/journal.pmed.1002164 27802277 PMC5089547

[7] Mayasari D Indriyani R Ikkom B KF Faktor Risiko dan Pencegahannya stunting, risk factors and prevention J Kesehat dan Agromedicine 2018 5 540 545

[8] Millward D J Nutrition, infection and stunting: the roles of deficiencies of individual nutrients and foods, and of inflammation, as determinants of reduced linear growth of children Nutr Res Rev 2017 30 01 50 72 28112064 10.1017/S0954422416000238

[9] Vieira K A Bastos C M Vitor M GC Use of low-level laser therapy on children aged 1 to 5 years with energy-protein malnutrition: a clinical trial Medicine (Baltimore) 2018 97 17 e0538 10.1097/MD.0000000000010538 29703031 PMC5944562

[10] Lynch R JM The primary and mixed dentition, post-eruptive enamel maturation and dental caries: a review Int Dent J 2013 63 02 3 13 24283279 10.1111/idj.12074 PMC9375027

[11] Reddy V Protein energy malnutrition; an overview Prog Clin Biol Res 1981 77 01 227 235 6174991

[12] Singh N Bansal K Chopra R Kamal C Dharmani K Association of nutritional status on salivary flow rate, dental caries status and eruption pattern in pediatric population in India Indian Journal of Dental Sci. 2018 10 02 78 82

[13] Raval H Rao A Chauhan H Estimation of serum albumin and serum total protein levels in children with protein energy malnutrition Int J Paediatr Geriatri 2020 3 01 76 78

[14] Soesilawati P Notopuro H Yuliati Y Ariani M D Alwino Bayu Firdauzy M The role of salivary sIgA as protection for dental caries activity in Indonesian children Clin Cosmet Investig Dent 2019 11 291 295 10.2147/CCIDE.S194865 PMC6730538 31564987

[15] Choukroune C Tooth eruption disorders associated with systemic and genetic diseases: clinical guide J Dentofac Anom Orthod 2017 20 04 402 10.1051/odfen/2018129

[16] Gentile C Chiarelli F Rickets in children: an update Biomedicines 2021 9 07 738 10.3390/biomedicines9070738 34199067 PMC8301330

[17] Khurshid Z Zafar M Khan E Mali M Latif M Human saliva can be a diagnostic tool for Zika virus detection J Infect Public Health 2019 12 05 601 604 31129010 10.1016/j.jiph.2019.05.004

[18] Zhou J Tang L Wang J S Assessment of the adverse impacts of aflatoxin B _1_ on gut-microbiota dependent metabolism in F344 rats Chemosphere 2019 217 618 628 30447610 10.1016/j.chemosphere.2018.11.044

[19] Matsuzaki K Sugimoto N Islam R Salivary immunoglobulin a secretion and polymeric ig receptor expression in the submandibular glands are enhanced in heat-acclimated rats Int J Mol Sci 2020 21 03 E815 10.3390/ijms21030815 PMC7037029 32012687

[20] Alhajj M Farhana A Enzyme Linked Immunosorbent Assay. [Updated 2022 Feb 2] Treasure Island (FL) StatPearls Publishing2022 Jan-. Accessed July 3, 2022 from:https://www.ncbi.nlm.nih.gov/books/NBK555922/32310382

[21] pour Nouroozi R Determination of protein concentration using Bradford microplate protein quantification assay Int. Electron. J. Med 2015 4 01 11 17

[22] Benkerroum N Aflatoxins: producing-molds, structure, health issues and incidence in Southeast Asian and Sub-Saharan African countries Int J Environ Res Public Health 2020 17 04 1215 10.3390/ijerph17041215 32070028 PMC7068566

[23] Liew W-P-P Mohd-Redzwan S Than L TL Gut microbiota profiling of aflatoxin B1-induced rats treated with *Lactobacillus casei* Shirota Toxins (Basel) 2019 11 01 49 10.3390/toxins11010049 30658400 PMC6357033

[24] Alamu E O Gondwe T Akello J Maziya-Dixon B Mukanga M Relationship between serum aflatoxin concentrations and the nutritional status of children aged 6-24 months from Zambia Int J Food Sci Nutr 2020 71 05 593 603 31718342 10.1080/09637486.2019.1689547

[25] Nabwera H M Bernstein R M Agbla S C Hormonal correlates and predictors of nutritional recovery in malnourished African children J Trop Pediatr 2018 64 05 364 372 29092084 10.1093/tropej/fmx075 PMC6166213

[26] Abdullahi S M Yakubu A M Bugaje M A Akuyam S M Serum total protein and albumin levels among malnourished children aged 6- 59 months in Zaria Niger J Paediatric 2018 45 01 15 18

[27] Rytter M J Namusoke H Ritz C Correlates of thymus size and changes during treatment of children with severe acute malnutrition: a cohort study BMC Pediatr 2017 17 01 70 10.1186/s12887-017-0821-0 28288591 PMC5348758

[28] Ağırdil Y The growth plate: a physiologic overview EFORT Open Rev 2020 5 08 498 507 32953135 10.1302/2058-5241.5.190088 PMC7484711

[29] Campisi S C Carducci B Söder O Bhutta Z A The Intricate Relationship between Chronic Undernutrition, Impaired Linear Growth and Delayed Puberty: Is 'catch-up' growth possible during adolescence? Off Res - Innocenti Work Pap 2018WP-2018–12(July):1–31. https://www.unicef-irc.org/publications/pdf/WP%202018%20-%2012.pdf. Accessed July 3, 2022

[30] Hajri T Angamarca-Armijos V Caceres L Prevalence of stunting and obesity in Ecuador: a systematic review Public Health Nutr 2021 24 08 2259 2272 32723419 10.1017/S1368980020002049 PMC10195486

[31] Al-Taiar A Alqaoud N Sharaf Alddin R Stunting and combined overweight with stunting among schoolchildren in Kuwait: trends over a 13-year period Med Princ Pract 2021 30 06 515 521 34348312 10.1159/000518533 PMC8739943

[32] Knuth M M Mahapatra D Jima D Vitamin D deficiency serves as a precursor to stunted growth and central adiposity in zebrafish Sci Rep 2020 10 01 16032 10.1038/s41598-020-72622-2 32994480 PMC7524799

